# High Resolution Electrocardiography

**Published:** 2002-04-01

**Authors:** S Narayanaswamy

**Affiliations:** Rajal Biotechnologies, Fremont, CA, USA

## Abstract

Over the past decade, significant advances were made in the research, diagnosis, and treatment of cardiovascular diseases. Such progress was in every sphere of cardiology that includes non-invasive, minimally invasive, and invasive technologies. Interpretive electrocardiography, cardiac pacemakers, cardiac stents, and angioplasty are some areas where the progress has been significant. Non-invasive methods of diagnosis of cardiac disorders involve digital recording of cardiac signals at the body surface (chest) and subsequent computerized analysis. Such methods and instruments provide a vital first step to the diagnosis of the heart without involving surgical procedures. One such non-invasive field is High Resolution Electrocardiography (HRECG). A high-resolution electrocardiogram detects very low amplitude signals in the ventricles called 'Late Potentials' in patients with abnormal heart conditions. A standard electrocardiogram cannot detect these signals. The presence of late potentials is widely accepted to have prognostic significance in patients after Acute Myocardial Infarction (AMI)

High Resolution Electrocardiography enhances the diagnostic capabilities of ECGs. This article describes the principles involved in HRECG and the techniques that are employed to derive such superior diagnostic capabilities. The use of these techniques may lead to more discoveries in the causes of cardiac disorders and improved drug discoveries to combat such conditions.

## Introduction

The Electrocardiogram (ECG) is a graphical representation of the electrical potentials generated by the heart. Such electrical signals are recorded via ECG leads placed on the body surface. Based on the resolution of the digital recording of analog ECG signals, the instruments and techniques may be categorized into two types: 1) Low-resolution (or standard) ECG, and 2) High-Resolution Electrocardiogram (HRECG). A standard 12-Lead ECG is a typical example of a widely used low-resolution instrument that records 9 seconds of cardiac data. A Signal-Averaged Electrocardiogram (SAECG) is a typical example of a High-Resolution Electrocardiogram. The latter signifies recent innovations in advanced ECG analysis and diagnosis. These instruments record ventricular ECG signals of very low magnitudes called 'Ventricular Late Potentials' (VLP) by averaging a number of signals. The presence of VLPs is indicative of increased risk for subsequent occurrence of arrhythmic events, mainly sustained ventricular tachycardia [1-3].

## Cardiac Late Potentials

Cardiac late potentials are low amplitude signals that occur in the ventricles. Also called Ventricular Late Potentials (VLPs), these signals are caused by slow or delayed conduction of the cardiac activation sequence. Under certain abnormal conditions, there may be small regions of the ventricles within a diseased or ischemic region that generate such delayed conduction. This results in depolarization signals that prolong past the refractory period of surrounding tissues and re-excite the ventricles [4]. This re-excitement is known as 'reentry'. Reentry is believed to be a key factor that causes VLPs.

### Late Potentials and the Electrocardiogram

The electrocardiogram represents the depolarization and repolarization of the major chambers of the heart. Under normal conditions, the ventricular regions to be activated coincide with the end of the QRS complex. However, in conditions of reentry, VLPs appear a few microvolts extending well beyond the terminal portion of the QRS in the ECG.

Due to their very low magnitudes, late potentials are not visible in a standard ECG. Moreover, factors such as increased distance of the body surface electrodes from the heart, and inherent noise in patients make identification of VLPs beyond the resolution limits of a standard ECG. As a result, high-resolution recording techniques and computerized ECG processing are necessary for detection of late potentials. Such ECG signal processing includes techniques to improve the signal-to-noise ratio (SNR). One widely used technique to improve the SNR of ECG signals for the detection of late potentials is signal averaging.

### Signal Averaging

Signal Averaging is a signal processing technique that is applied to signals that are periodic. Every signal inherently has two components: 1) signal, and 2) noise. By adding many signals, the overall noise component of the signal sum will decrease while the desired signal component remains unchanged [5]. Since ECG signals are periodic in nature, inherent noise in the signals can be minimized thus enhancing the signal component in the process. This signal enhancement will improve the resolution of the overall signal especially the detection of late potentials. Figure 1a and b show an ECG signal and a SAECG signal of the same patient. The noise components are significantly reduced in the SAECG signal

## HRECG Instrument

**Figure 2:**
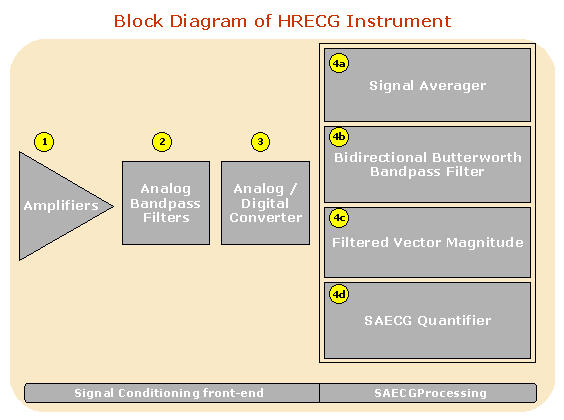
HRECG block diagram

Figure 2 illustrates the block diagram of a typical HRECG instrument. A HRECG instrument may be divided into two major parts: 1) signal acquisition front-end, and 2) computerized SAECG processing. The components of each part are described in greater detail in the following sections.

### Components

As illustrated in Figure 2, an HRECG instrument consists of 4 key components: 1) Amplifiers, 2) Bandpass filters, 3) Analog/Digital converter, and 4) SAECG Processor. The SAECG Processor may in turn be functionally divided into the following components: a) Signal Averager, b) Bidirectional Bandpass Filter, c) Filtered Vector Magnitude, and d) SAECG Quantifier. In addition, the instrument includes 7 ECG leads. These leads are bipolar, orthogonal electrodes comprising X+, X-, Y+, Y-, Z+, Z-, and ground placed in a particular fashion on the body surface [6]. These electrodes are usually referred to as XYZ leads.

#### Amplifiers

ECG signals are acquired using precision instrumentation amplifiers. This electronic circuitry is a critical part in the signal conditioning front-end, as noise is a serious hindrance to high-resolution signal acquisition. These devices combine the positive and negative components of a signal (X+, X-) to yield a differential signal with reduced noise.

#### Bandpass Filter

Most of the energy in ECG signals are concentrated in the bandwidth between 1Hz - 80Hz. This analog base-bandwidth is typical to standard ECGs. However, for HRECG signal acquisition, a broad bandwidth of 0.05Hz - 300Hz is considered in order to acquire as many signal frequencies as possible. As a result, the signals are sampled at very high frequencies. Typical minimum sampling frequencies are ≥ 1000Hz (1KHz).

#### Analog / Digital Converter

An analog-to-digital converter (ADC) allows the digitization of an analog signal. After conversion, analog signals that are continuous in nature are represented as a digital stream of data. This digital data is stored in memory as binary bits. ADCs in HRECG instruments should have a minimum of 12-bit resolution i.e. every data point is 12-bits of digital data. Once converted, the signal representation is transformed from the analog domain to the digital domain.

#### SAECG Processor

The SAECG processing functionality is digitally implemented either through hardware components or through software via computerized processing. In hardware, a general purpose Digital Signal Processor (DSP) can perform all the 4 key SAECG functional blocks. On the other hand, each SAECG block can be implemented in software. Each of these functions is described in greater detail below.

##### Signal Averager

Signal averaging is the crux of an HRECG instrument. As explained below, a signal averager computes the average value of each data point in a cardiac signal after averaging many such signals. Consequently, the resultant average results in minimized noise, and allows for high resolution processing of the signal average.

##### Bidirectional Bandpass Filter

Signal averaging may introduce ST segments shifts. Filters are used on a signal average to eliminate any bias especially in the terminal QRS and ST segment regions. Typically a recommended bandpass filter is a bi-directional Butterworth implementation [7]. This implementation is a 2nd order low-pass filter and a 4th order highpass filter with cutoff frequencies of 250Hz and 400Hz respectively. These filters inherently cause ringing in the signal. Consequently, artifacts may be introduced in the regions of interest, which may be mistaken for late potentials. A bi-directional implementation of the Butterworth filter shifts this ringing into regions of the QRS without distorting the terminal QRS endpoints [8].

##### Filtered Vector Magnitude

 The filtered vector magnitude is computed as (X2 + Y2 + Z2)1/2. The results of this computation are the basis for quantifying the SAECG parameters for late potential identification.

##### SAECG Quantifier

The SAECG quantifier determines the HRECG parameters that identify late potentials. The criteria for late potential identification are described below. The SAECG parameters are based on these criteria.

## HRECG Parameters: Criteria to Identify Late Potentials

The task force committee of the European Society of Cardiology, the American Heart Association and the American College of Cardiology [9] suggested a representative criteria for the identification of late potentials. This criteria is defined in Table 1.

### A High Resolution Electrocardiogram

**Figure 3:**
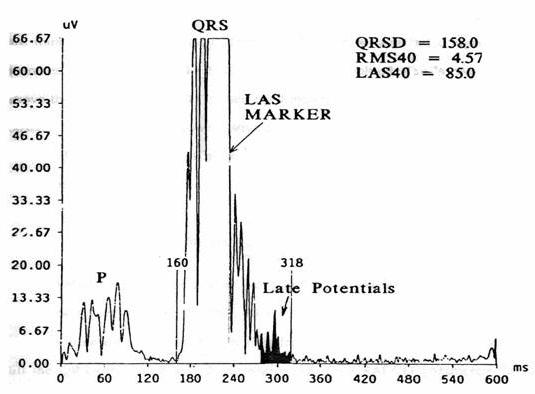
HRECG of a patient with positive late potentials

A high-resolution electrocardiogram is aimed at identifying late potentials. It graphically represents the filtered Vector Magnitude of the XYZ leads. Figure 3 illustrates a high-resolution electrocardiogram of a patient with positive late potentials.

### Standard ECG vs HRECG

As described in the sections above, a HRECG differs from a standard ECG in several ways. Table 2 summarizes some of the key differences between the two.

## Clinical Significance

The ability to detect cardiac late potentials in a High Resolution ECG has significant clinical implications. What was originally undetectable is no longer the case and is primarily due to the advancements in non-invasive signal acquisition and processing techniques such as signal averaging. The detection of late potentials in patients has prognostic significance especially in patients with acute myocardial infarction. The presence of ventricular late potentials in patients with acute MI is an indicator of risk from subsequent MI or sudden cardiac death. Earlier this classification and subsequent diagnosis was possible only via invasive or minimally invasive techniques. A SAECG study is accepted as a standard procedure in the United States with due reimbursement from Medicare.

## Conclusion

 High Resolution Electrocardiography has serious research and clinical significance. The techniques used in SAECG can be applied to a variety of other research topics in order to better study the mechanisms of cardiac abnormalities that manifest on the body surface as abnormal signals, for example: premature ventricular contractions.

## Figures and Tables

**Figure 1a:**
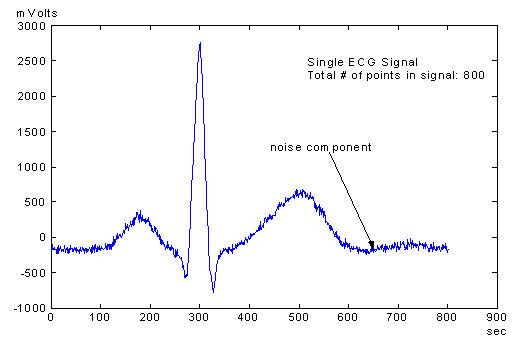
An ECG signal with noise components

**Figure 1b:**
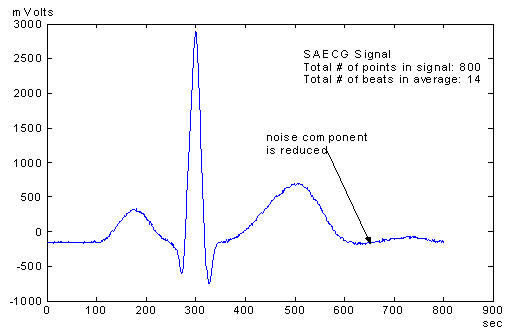
A SAECG signal

**Table 1:**
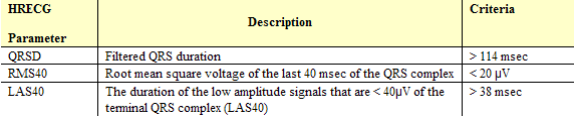
Criteria for Late Potential Identification

**Table 2:**
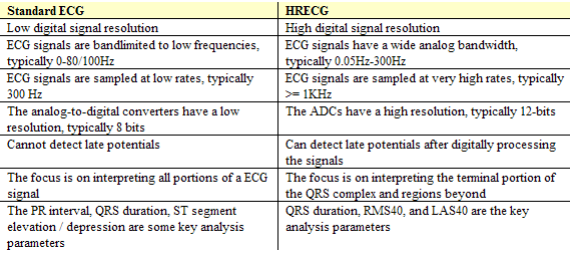
Some differences between Standard ECG and HRECG
